# HBV-associated DLBCL of poor prognosis: advance in pathogenesis, immunity and therapy

**DOI:** 10.3389/fimmu.2023.1216610

**Published:** 2023-07-07

**Authors:** Xin Wan, Ken H. Young, Ou Bai

**Affiliations:** ^1^ Department of Hematology, The First Hospital of Jilin University, Changchun, Jilin, China; ^2^ Department of Hematopathology, Duke Cancer Institute, Duke University Medical Center, Durham, NC, United States

**Keywords:** hepatitis B virus, DLBCL, epigenetics, prognosis, chemo-resistance

## Abstract

Advanced studies have shown a biological correlation between hepatitis B virus (HBV) and B-cell lymphoma, especially diffuse large B-cell lymphoma (DLBCL). Patients with DLBCL infected with HBV (HBV-associated DLBCL) are clinically characterized by an advanced clinical stage, poor response to front-line immunochemotherapy regimens, and worse clinical prognosis. HBV-associated DLBCL often exhibits abnormal activation of the nuclear factor kappa B pathway as well as mutations in oncogenes, including *Myc* and *BCL-6*. Currently, there is no consensus on any specific and effective treatment for HBV-associated DLBCL. Therefore, in this review, we comprehensively and mechanistically analyzed the natural history of HBV infection and immunity, including HBV-mediated oncogenes, immune escape, epigenetic alterations, dysregulated signaling pathways, and potential therapeutic approaches for HBV-associated DLBCL. We hope that an improved understanding of the biology of HBV-associated DLBCL would lead to the development of novel therapeutic approaches, enhance the number of effective clinical trials, and improve the prognosis of this disease.

## 1 Introduction

Diffuse large B-cell lymphoma (DLBCL) is the most common type of B cell lymphoma, comprising 30%–40% of non-Hodgkin lymphomas (NHL). Most patients with DLBCL can be cured using current front-line immunochemotherapy regimens based on a combination of anthracyclines and anti-CD20 antibodies, such as R-CHOP; however, 30%–40% of patients have disease relapse or are refractory to first-line treatment, meaning that DLBCL is a possibly heterogeneous disease (1). Over the past few decades, considerable efforts have been made to decipher the molecular basis of this heterogeneity. First, according to the gene-expression profiles of the cell of origin, DLBCL has been divided into three subtypes, namely activated-B-cell-like (ABC), germinal-center B-cell-like (GCB), and an unclassified group; patients with GCB DLBCL have been reported to have a significantly better overall survival (OS) than those with ABC DLBCL (2). Moreover, DLBCLs harboring Myc, BLC-2, and/or BCL-6 translocations are called “double-hit” lymphoma (DHL) or “triple-hit” lymphoma (THL), which have a poor prognosis. In the recent 5^th^ edition of the World Health Organization classification of lymphoid neoplasms, the DHL/THL category is recognized as “high-grade B-cell” lymphoma owing to the rearrangements of Myc and BCL-2 (3). However, there are certain disadvantages to first-line chemotherapy of DLBCL, including hepatitis B virus (HBV) infection (HBV-associated DLBCL). In this review, we focus on whether HBV infection worsens the prognosis of patients with HBV-associated DLBCL through the aforementioned related molecules and/or cell signaling.

## 2 HBV-associated DLBCL with poor prognosis

### 2.1 Clinical features

Many studies have validated that HBV can lead to other kinds of cancers besides hepatocellular carcinoma (HCC), including gastrointestinal tumors and B-cell non-Hodgkin’s lymphoma (B-NHL), in Asia, Africa and Western countries (4–7). Moreover, among patients with B-NHL, DLBCL has been reported to have a strong and more significant association with HBV infection (8–11) compared to that with indolent lymphoma (12–14); for instance, a study from west Africa showed that the prevalence of HBV-associated DLBCL is 14.3% (5). Moreover, a previous study of ours showed that the incidence of HBV-associated DLBCL is 13.2% (15), meaning that HBV infection is closely related to DLBCL.

HBV-associated DLBCL is characterized by a high prevalence among younger individuals, a high incidence at advanced clinical stages, mainly involving the peritoneal lymph nodes and spleen, and a relatively poor prognosis (16).Our previous study showed that patients with DLBCL and HBV infection had poor prognosis (three-year OS: HBsAg-negative patients, 90.6% *vs.* patients with HBV-associated DLBCL 54.5%) (17). Cheng et al. (18) also found that patients with HBV-associated DLBCL had a lower overall response rate (ORR: 76.5% *vs.* 85.5%, *p*=0.043), poorer 5-year OS rate (57.2% *vs.* 73.5%, *p <*0.001), and a shorter 5-year progression-free survival (PFS: 47.2% *vs.* 60.7%, *p*=0.013) than HBsAg-negative patients. HBsAg-positivity is an independent adverse prognostic factor for HBV-associated DLBCL (18, 19). It is also the main factor leading to the poor efficacy of immunochemotherapy, including R-CHOP regimens. Accordingly, HBV infection is closely associated with DLBCL and is a crucial factor leading to poor prognosis (17, 20, 21). Huang et al. indicated that antiviral agents, entecavir, combined with chemotherapy could improve the prognosis of patients with HBV-associated DLBCL (median OS not reached *vs.* 35.61 months) (22). In this review, we focus on the dysregulation in molecules related to the poor prognosis of HBV-associated DLBCL, including oncogenes, immune escape, epigenetic modification, and dysregulated signaling pathways, as well as chemo-resistance and potential therapeutic approaches.

### 2.2 Genomic structure of HBV, immunity, and dysregulation

**Figure 1:**
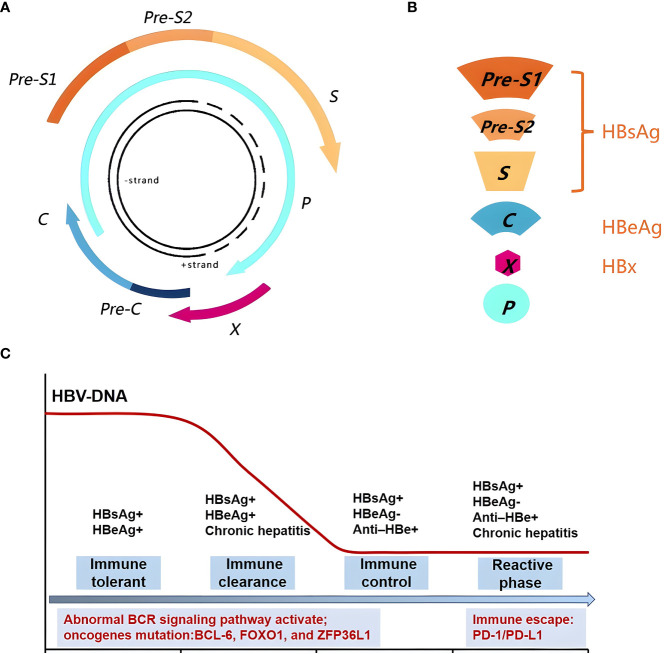
Genomic structure of HBV and relationship with the body immune function. **(A)** The HBV Genomic structure. **(B)** HBV gene code associated protein. **(C)** The natural history of HBV infection has four phases, including immune tolerant, immune clearance, immune-control and reactive phase, could activate the abnormal BCR signaling pathway and oncogenes mutation. The reactive phase is associated with immune escape.

The HBV genome encompasses four open overlapping reading frames (ORFs)-pre-C/C (pre-core/core), pre-S/S (surface proteins), X (transcriptional co-activator), and P (DNA polymerase) (23) (**Figure 1A**). The preS1, preS2, and S genes mainly code HBsAg; pre-C/C code HBeAg; and the HBV *X* gene codes the HBx protein (**Figure 1B**). After HBV infection, the human body initially carries the virus for a period of time and subsequently expresses the relevant antigens, including HBsAg and HBeAg, thereby eliciting immune response by stimulating the B-cell receptor (BCR) and subsequently activating the abnormal BCR signaling pathway. B-cells produce antibodies HBsAb, HBeAb, and HBcAb to block further infection and support effective viral clearance (24). The natural history of HBV infection has four phases, including immune tolerance, immune clearance, immune-control, and reactive phase (25–27). (1) The immune tolerant phase is characterized by HBeAg seropositivity with high viral loads (>10^6-7^ IU/mL) but near-normal liver histology. (2) The immune clearance phase is characterized by HBeAg positivity with a declining serum HBV-DNA level and active inflammation in the liver; this phase may eventually lead to HBV-DNA seroclearance and the seroconversion of HBeAg to its antibody (HBeAb) in most patients. (3) In the immune-control phase, following HBeAg seroconversion, most patients enter the immune-control phase with low serum HBV-DNA (<2000 IU/mL). (4) The reactive phase is characterized by HBeAg negativity with HBeAb positivity, detectable serum HBV-DNA levels, and active inflammation in the liver; this phase is associated with immune escape (**Figure 1C**).

**Figure 2:**
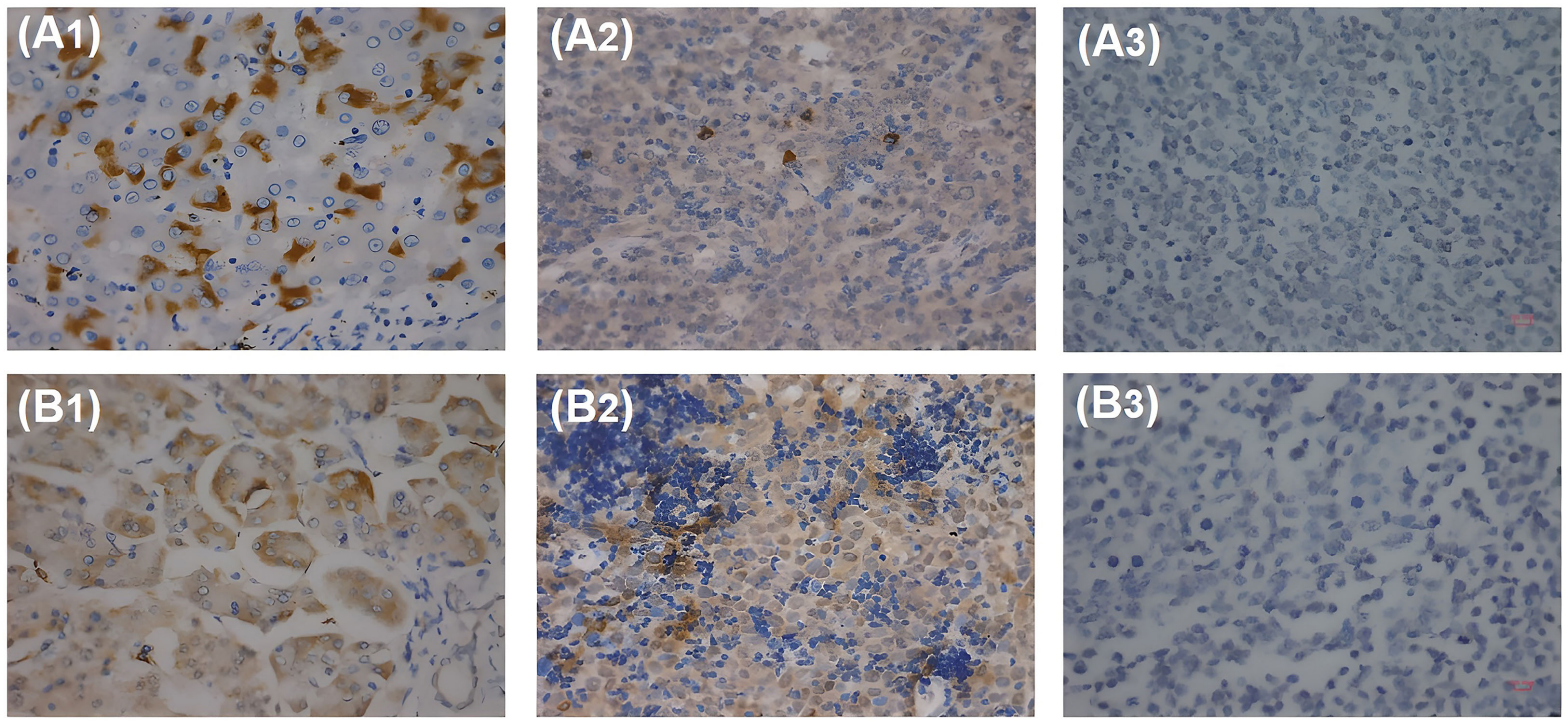
HBx and pre-S2 detected in patients with HBV-associated DLBCL with IHC. **(A1)** HBx antigen expressed in HBV-associated hepatocytes. **(A2)** HBx antigen expressed in HBV-associated DLBCL lymphoma cells. **(A3)** HBx antigen cannot be detected in HBV-negative DLBCL lymphoma cells. **(B1)** Pre-S2 antigen expressed in HBV-associated hepatocytes. **(B2)** Pre-S2 antigen expressed in HBV-associated DLBCL lymphoma cells. **(B3)** Pre-S2 antigen cannot be detected in HBV-negative DLBCL lymphoma cells.

As for genes, *HBx* is the most frequently integrated viral gene following HBV infection, which plays a critical role in tumor pathogenesis (28). Studies have reported that HBx was detected in the tissues of patients with HBV-associated DLBCL (29, 30). Our previous study has also shown that HBx was detected in tissues from patients with HBV-associated DLBCL and that HBx expression was correlated with c-Myc expression (31) (**Figure 2**).

#### 2.2.1 Crucial oncogene mutation and corresponding cell signaling pathways

**Figure 3:**
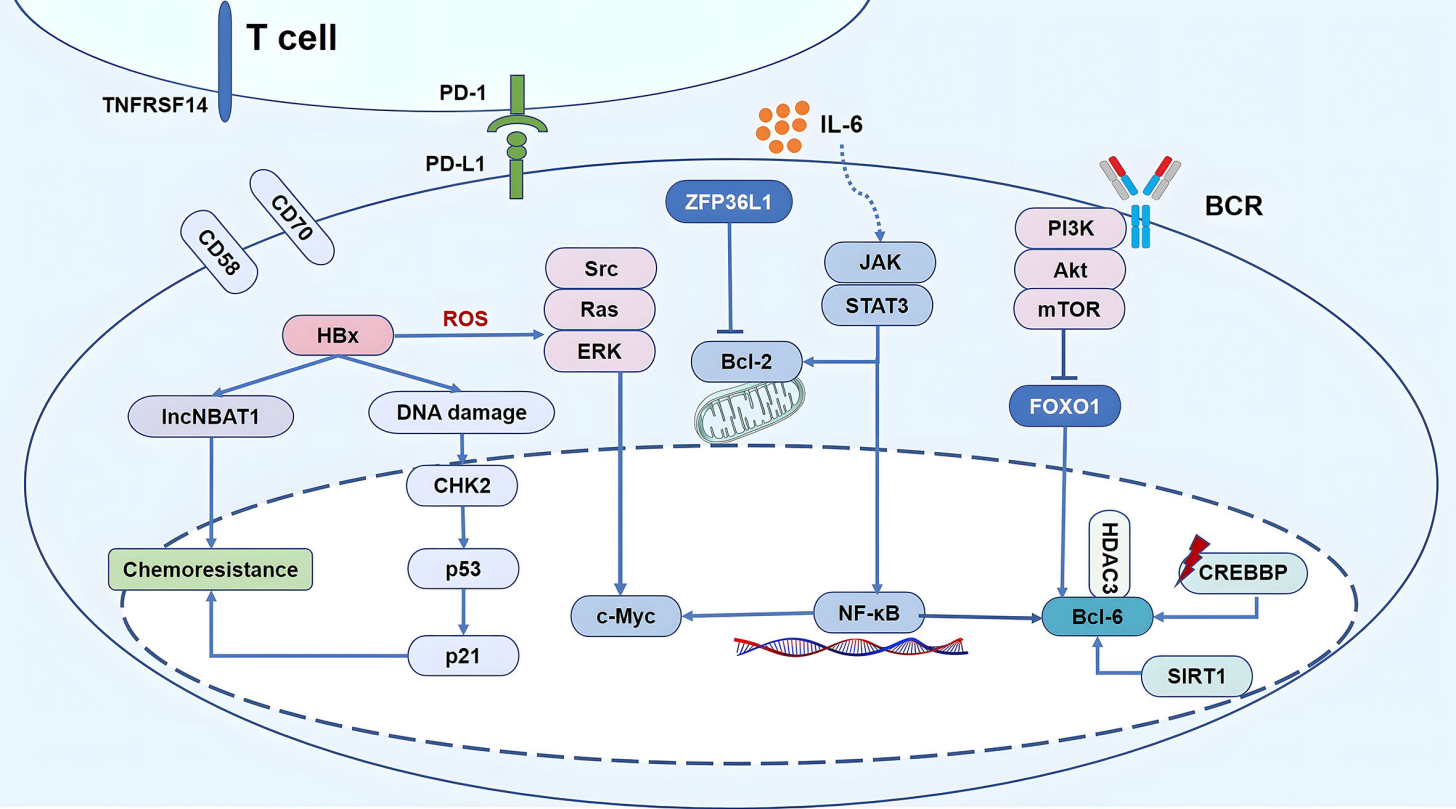
Schematic diagram of HBV-mediated oncogenic signaling pathway activation, epigenetic modification, immune escape and chemo-resistance in HBV-associated DLBCL. **1)** Replication of the virus leads to abnormal host epigenetic regulation, accompanied by CREBBP mutations up-regulation of SIRT1, inducing the up-regulation of deacetylatation levels, resulting in imbalance of CREBBP-BCL-6/HDAC3 axis, finally increasing the activity of BCL-6 as proto–oncogenes. Gene expression signature of HBV-associated DLBCL was contributed by the enrichment of genes regulated by *BCL-6, FOXO1*, and *ZFP36L1* and involved in signaling pathways, including BCR, PI3K, JAK/STAT, and NF-κB. In addition, HBx simultaneously activates the Src/Ras/ERK pathway, promoting proliferation and anti-apoptosis owing to the accumulation of ROS and endoplasmic reticulum stress. **2)** Tumor-infiltrating immune cells CD8+ T cells are in a state of functional depletion, highly expressed PD-1. The activation of the PD-1/PD-L1 cell signaling pathway can suppress the function of the immune system. CD70, TNFRSF14, and CD58 were mainly mutated in HBV-associated DLBCL, led to a decrease in T cell infusion in tumor microenvironment. **3)** HBx over expression significantly reduces the activation of CHK2 signals induced by S-period blockers and p53 in HBV-associated DLBCL, thereby reducing the sensitivity of chemotherapy drug; HBx directly up-regulated the expression of lncNBAT1, reduced the sensitivity of DLBCL cells to chemotherapeutic agents that induced S phase arrest.

Whole exome sequencing/whole genome sequencing (WES/WGS) showed that HBV infection was associated with DLBCL. Studies indicated several mutations in genes related to the AID/APOBEC enzymes in patients with HBV-associated DLBCL (32, 33). Ren et al. (32) detected the genes mutation in 275 patients of DLBCL, including 20% patients with HBsAg positive (56/275). And the tumor biopsy specimens of 229 patients were taken at diagnosis, and 46 were taken at relapse. The quantitation of viral DNA was performed in 44 HBsAg positive patients. Fourteen genes were confirmed to be preferentially mutated in the HBV infection group, including *KLF2, TMSB4X, CD70, BCL-6, FAS, TNFRSF14, UBE2A, CD58, SGK1, ZFP36L1, CXCR4, FOXO1, CSK*, and *MSL2*. In these genes, the enrichment of genes regulated by *BCL-6, FOXO1*, and *ZFP36L1* and involved in signaling pathways, including BCR, janus kinase/signal transducer and activator of transcription (JAK/STAT), and nuclear factor kappa B (NF-κB), contributed to the gene expression signature (**Figure 3**).

BCR signaling activates downstream oncogenic pathways, including NF-κB or PI3K (34). The PI3K-Akt-mTOR signaling cascade is known to be dysregulated and represents a major regulator of cell survival-proliferation (35). NF-κB regulates the proliferation of B cells and promotes the expression of proto-oncogenes (*Myc* and *BCL-6*) and cellular anti-apoptotic protein (BCL-2), as well as the secretion of cytokines, including tumour Necrosis Factor alpha (TNFα), lymphotoxin-α, and interleukin-6; it further positively regulates the JAK/STAT3 pathway and cell proliferation and promotes tumorigenesis. As a key transcription factor, BCL-6 plays a crucial role in the clearance of HBV (36). Furthermore, BCL-6 is a crucial gene of HBV-associated DLBCL. There was no significant difference between the HBsAg positive and HBsAg negative groups in the percentage of GCB *vs.* non-GCB patients. The incidence of GCB DLBCL was 37.5% *vs.* 38.1%, respectively; non-GCB was 62.5% *vs.* 61.9%, respectively (32). Zhang et al. showed that HBV was important in DHL/THL DLBCL and that the incidence of Myc/BCL*-*6 DHL was higher than that of Myc/BCL*-*2 DHL with HBV infection (37). A previous study of ours showed that the rate of Myc rearrangements in patients with HBV-associated DLBCL was significantly higher than that in HBV-free individuals (38). Moreover, HBx simultaneously activated the Src/Ras/ERK pathway, promoting proliferation and anti-apoptosis owing to the accumulation of ROS and endoplasmic reticulum stress (39). Therefore, HBV-associated DLBCL is involved in the signaling pathways, including BCR, NF-κB, and JAK/STAT3, and further dysregulates oncogenes, including BCL-6, Myc, which are associated with poor prognosis. Moreover, following HBV infection, the human body activates the abnormal BCR signaling pathway, which could be more directly related to HBV-associated DLBCL.

#### 2.2.2 Immune escape

In the reactive phase, HBV could not be completely eliminated and induce immune escape, which is associated with the poor survival of patients with HBV-associated DLBCL. During chronic HBV infections, virus-specific T-cells appear deeply exhausted. Both CD8 and CD4 T-cells up-regulate co-inhibitory receptors, which can inhibit T-cell function following the cross-linking of their ligands, entailing T-cell exhaustion and tumor escape. For instance, the programmed cell death 1/programmed cell death ligand 1 (PD-1/PD-L1) cell signaling pathway is closely related to the functioning of the immune system, which is currently a research hotspot in the field of tumor immunotherapy. The activation of the PD-1/PD-L1 cell signaling pathway can suppress the function of the immune system and contribute to the immune escape of cancer cells (40). Our previous study showed that the incidence of PD-1 expression among patients with HBV-associated DLBCL was 4.3-fold higher than that among HBV-free individuals (40.0% *vs.* 9.4%; *p*=0.010). Moreover, the median OS and PFS were the worst in PD-1-positive patients with HBV-associated DLBCL. These results indicate that the dismal prognosis of patients with HBV-associated DLBCL may be related to the high rate of PD1 expression (15) (**Figure 3**).

Moreover, the number of CD4-positive lymphocytes has been reported to decrease significantly after chemotherapy in patients with HBV-associated DLBCL, and the CD4:CD8 ratio decreased for a longer time in the aforementioned population than in HBV-free individuals, elucidating the reason behind the poor prognosis of patients with HBV-associated: The host immune system weakened the tumor surveillance effect, leading to rapid disease recurrence and poor prognosis (41). Moreover, Ren et al. (32) showed that CD70, TNFRSF14, and CD58 were the main mutated genes among patients with HBV-associated DLBCL, leading to a decrease in T-cell infusion in the tumor microenvironment and thereby weakening tumor immune surveillance and accelerating their escape.

In summary, the continuous high expression of multiple co-inhibitory molecules is the key cause of the depletion of HBV-specific T-cell function. Blocking these molecules can significantly restore HBV-specific T cell function and subsequently remove the virus *in vivo*. Therefore, the blocking of co-stimulatory molecules might represent a novel strategy for the clinical treatment of HBV infection.

#### 2.2.3 Noncoding RNAs and epigenetic modification

HBx does not bind to DNA, and HBx-activated RNA polymerase (pol) I-, II-, and III-dependent promoters directly interact with some transcription factors and stimulate signal-transduction pathways. The HBx protein can initiate epigenetic modifications to dysregulate noncoding RNAs expression, which, consequently, can regulate downstream epigenetic changes throughout the pathogensis of HBV-associated DLBCL. Bruni et al. (42) found that patients with HBV-associated indolent B-NHL have dysregulated miRNA. Chen et al. (43) also showed that this dysregulation of miRNAs was closely related to the proliferation and differentiation of B-cells. Of note, miRNAs regulate through histone deacetylase (HDAC) (44) or the NF-κB pathway (45). While HDAC inhibitors can break this negative regulation by promoting the expression of miR-34a (46). Several studies have shown that epigenetic modification play a significant role in patients with HBV-associated DLBCL, which have a higher frequency of the CREB-binding protein (CREBBP) mutations (27.2%) compared to the general population (47). CREBBP mainly regulates the activity of histone acetyl transferase (HAT), maintains the activity of HAT domain, regulates transcription factors, and affects immune regulation. However, CREBBP mutation (loss-of-function) inactivates the HAT domain and impairs acetylation-deacetylation balance, thereby increasing BCL-6 expression (**Figure 3**). Nevertheless, the selective inhibition of HDAC3 reverts the molecular phenotype of CREBBP mutations (48, 49). Moreover, SIRT1 plays an important role in the acetylation of B-cell germinal center and could lead to an imbalance of acetylation/deacetylation. SIRT1 could cause the deacetylation of BCL-6, p53, and other transcription factors (50) and up-regulate the expression of BCL-6 (51). BO et al. showed that chronic stimulation with HBsAg promoted the viability of the human B lymphoblastoid cell line through regulation of the SIRT1-NF-κB pathway (52) (**Figure 3**). In conclusion, noncoding RNAs and epigenetic regulation may play roles in the pathogenesis of HBV associated DLBCL.

#### 2.2.4 Chemo-resistance

HBx elicits a DNA damage response. It binds to p53 in the nucleus, inhibits the expression of p53-responsive genes, promotes the phosphorylation (inactivation) of Rb, lowers the activities of CDK inhibitors, promotes vascular regeneration, and inhibits p53 function through Rb, thereby inhibiting cell apoptosis and cell cycle (53). HBx itself can inhibit p53 from functioning as a cell cycle blocker, thereby promoting cell survival. Moreover, the overexpression of HBx significantly reduces the activation of CHK2 signals induced by S-period blockers and p53 and p21 in patients with HBV-associated DLBCL, consequently reducing the sensitivity of chemotherapy drugs and suggesting that CHK2 may be a potential factor in HBx-induced chemotherapy resistance (54). Li et al. (55) found that HBx directly up-regulated the expression of lncNBAT1, a long non-coding RNA (lncRNA) that is closely associated with the chemotherapy outcomes of patients with HBV-associated DLBCL. The up-regulation of lncNBAT1 reduced the sensitivity of DLBCL cells to the chemotherapeutic agents that induced S-phase arrest, whereas the knockdown of lncNBAT1 significantly relieved the chemo-resistance of HBx-expressing DLBCLs (**Figure 3**).

Therefore, patients with HBV-associated DLBCL have a poor prognosis owing to genomic instability, immune escape, epigenetic regulation, and chemo-resistance, leading to an uncontrolled cell cycle, obstruction of apoptosis, and increased frequency of gene mutations. Moreover, many signal pathways, including BCR, JAK/STAT3, and the NF-κB pathway, are involved in the pathogenesis of HBV-associated DLBCL (**Table 1**).

**Table 1:** Summary of molecular advance in HBV-associated DLBCL.

Genes			Functional pathway		References
*TP53, TP63*			P53 pathway		(32)
*BCL-6, CXCR4, KLF2, SGK1*			FOXO signaling, BCR signaling		(32)
***Immunomodulation/escape***			Targerts		References
			*CD70, TNFRSF14, CD58*		(32)
			*PD-1/PD-L1*		(15)
Epigenetic modification and noncoding RNA					
Types	Main regulators	Targets		Functional pathway	References
Histone Acethylation	CREBBP	*CDKN1A, CIITA, BCL-6*		Antigen presentation and TFH activation	(47–49)
Histone Deacetylation	SIRT1	*p53*, *BCL-6*		NF-κB signaling	(50–52)
miR-34a	SIRT1	*STAT3, HMGB1, BCL-2*		NF-κB signaling	(45, 46)
Chemo-resisitance					
CHK2		*p53, p21*		p53, CHK2 signaling	(54)
lncNBAT1	STAT1	*APOBEC3A*			(55)

## 3 Future prospects of targeted treatment to improve prognosis

**Figure 4:**
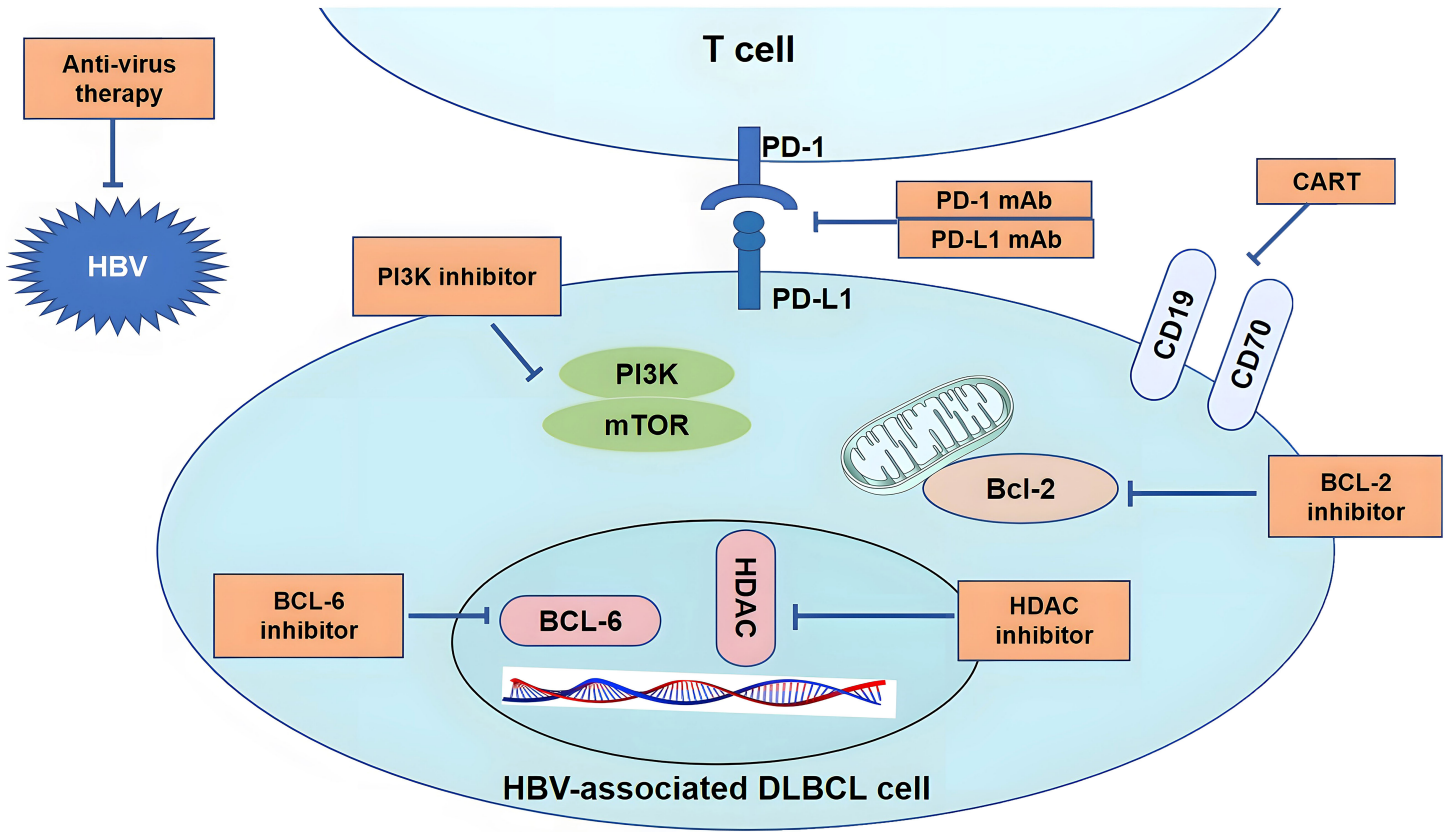
Therapeutic modulation of HBV infection and associated signaling pathways. Many therapeutic targets have been identified. (1) NAs suppress viral replication by inhibiting reverse transcription of pre-genomic RNA to HBV DNA in the cytoplasm. (2) small molecule kinase inhibitors, including epigenetic regulators, PI3K inhibitors, BCL-6 inhibitors and BCL-2 inhibitors. (3) immunotherapy, CAR-T, including CD19 and CD70 CAR-T; PD-1/PD-L1 mAb can activate T cells by regulating the PD-1/PD-L1 axis.

Patients with HBV-associated DLBCL respond poorly to current first-line immunochemotherapy. Therefore, novel therapeutic approaches for patients with HBV-associated DLBCL, including other virus-related lymphomas, should be considered (**Table 2**). Possible therapeutic approaches include: (1) anti-virus therapy; (2) small molecule kinase inhibitors, including epigenetic regulators, PI3K inhibitors, BCL-6 inhibitors, and BCL-2 inhibitors; and (3) immunotherapy, including CAR-T and immune-checkpoint inhibitors (**Figure 4**).

**Table 2:** Clinical ongoing trials in virus-related lymphoma.

Types	Treatment regimen	Results	NCT number
**EBV-positive lymphoma**	EBV-CTLs	CR:68%; 1y-OS: 88.9%	NCT01498484
	Brentuximab Vedotin	ORR:48% Median OS:15.6m	NCT02388490
	Sintilimab+R-CHOP	NA	NCT04181489
	Nanatinostat+ valganciclovir	NA	NCT05011058
**HIV-positive lymphoma**	Vorinostat + R-EPOCH	ORR:100% 1y-PFS:83%	NCT01193842
	Peripheral blood stem cells	NA	NCT00002221
	Brentuximab Vedotin + AVD	NA	NCT01771107
**HHV-positive lymphoma**	Oral Azacitidine	NA	NCT04799275
**HBV-positive lymphoma**	Chidamide	NA	NCT04661943

EBV, Epstein-Barr virus; HHV, Human Herpesvirus; HBV, Hepatitis B virus; R-CHOP, rituximab plus cyclophosphamide, doxorubicin, vincristine, and prednisone; R-EPOCH, rituximab plus etoposide, prednisone, vincristine, cyclophosphamide, and doxorubicin; AVD, adriamycin, vinblastine, and dacarbazine.

### 3.1 Anti-virus therapy

Nucleos(t)ide analogs (NAs) and interferons are two classes of antiviral agents currently licensed for the treatment of HBV infection. NAs, including entecavir, tenofovir disoproxil fumarate, and tenofovir alafenamide, suppress viral replication by inhibiting the reverse transcription of pre-genomic RNA to HBV DNA in the cytoplasm. Study indicated that entecavir combined with chemotherapy could improve the prognosis of patients with HBV-associated DLBCL (22). Moreover, Yamauchi et al. also showed that the prophylactic use of entecavir could reduce the occurrence of HBV reactivation-related hepatitis and mortality in patients with HBV-associated DLBCL receiving rituximab-containing chemotherapy (56).

### 3.2 Small molecule kinase inhibitors

#### 3.2.1 Epigenetic regulators

With advances in epigenetic research in recent years, the use of epigenetic regulators has gradually increased in clinical practice. Several clinical trials have confirmed the effectiveness and safety of HDAC inhibitors, including chidamide, in first-line consolidation and rescue therapy for the recurrence of lymphoma. Chidamide is a selective inhibitor of histone deacetylases (HDACs), which is mainly targeted at class I HDACs, and has a regulatory effect on the epigenetic function of tumor abnormalities. At our center, the First Hospital of Jilin University, Changchun, China, a clinical trial (**Table 2**; registered in www.clinicaltrials.gov under number NCT04661943) of chidamide maintenance treatment was conducted for patients with HBV-associated DLBCL. The inclusion criteria were achievement of complete response and complete response lasting for 1 year following systemic treatment before enrollment. The trial aimed at exploring the efficacy of epigenetic regulators in patients with HBV-associated lymphoma.

#### 3.2.2 Small molecule kinase inhibitors of cell signaling pathways

Such as PI3K, BCL-6, and BCL-2 inhibitors, and the immunomodulator lenalidomide have shown efficacy in treating recurrent and refractory DLBCL (57–60); however, clinical trials on the efficacy of these treatments for HBV-associated DLBCL are warranted.

### 3.3 Immunotherapy

#### 3.3.1 Chimeric antigen receptor-T cell therapy

CAR-T for the failure of multiline therapy of DLBCL has been demonstrated to have significant efficacy. Patients with HBV-associated DLBCL undergoing CAR-T cell therapy did not increase the risk of severe cytokine release syndrome (61). However, Yang et al. showed that patients with HBV-associated DLBCL receiving CD19-CAR-T cell therapy are at risk of HBV reactivation, especially in HBeAg-positive patients; the close monitoring of HBV-DNA levels and adequate antiviral prophylaxis are essential for the prevention of HBV reactivation (62). HBV-associated lymphoma presents immune escape-related gene mutations such as CD70, and targeted CD70 CAR-T may have potential efficacy.

#### 3.3.2 Immune-checkpoint inhibitors

PD-1 expression was higher in patients with HBV-associated DLBCL than in HBV-free individuals. Thus, immune-checkpoint inhibitors may have a satisfactory effect on patients with HBV-associated DLBCL. However, there is no standard first-line therapy, and clinical trials exploring specific treatment regimens for HBV-associated DLBCL are rare compared with those for other virus-related lymphomas. Further studies to explore more effective therapy regimens are warranted.

## 4 Conclusion

**Figure 5:**
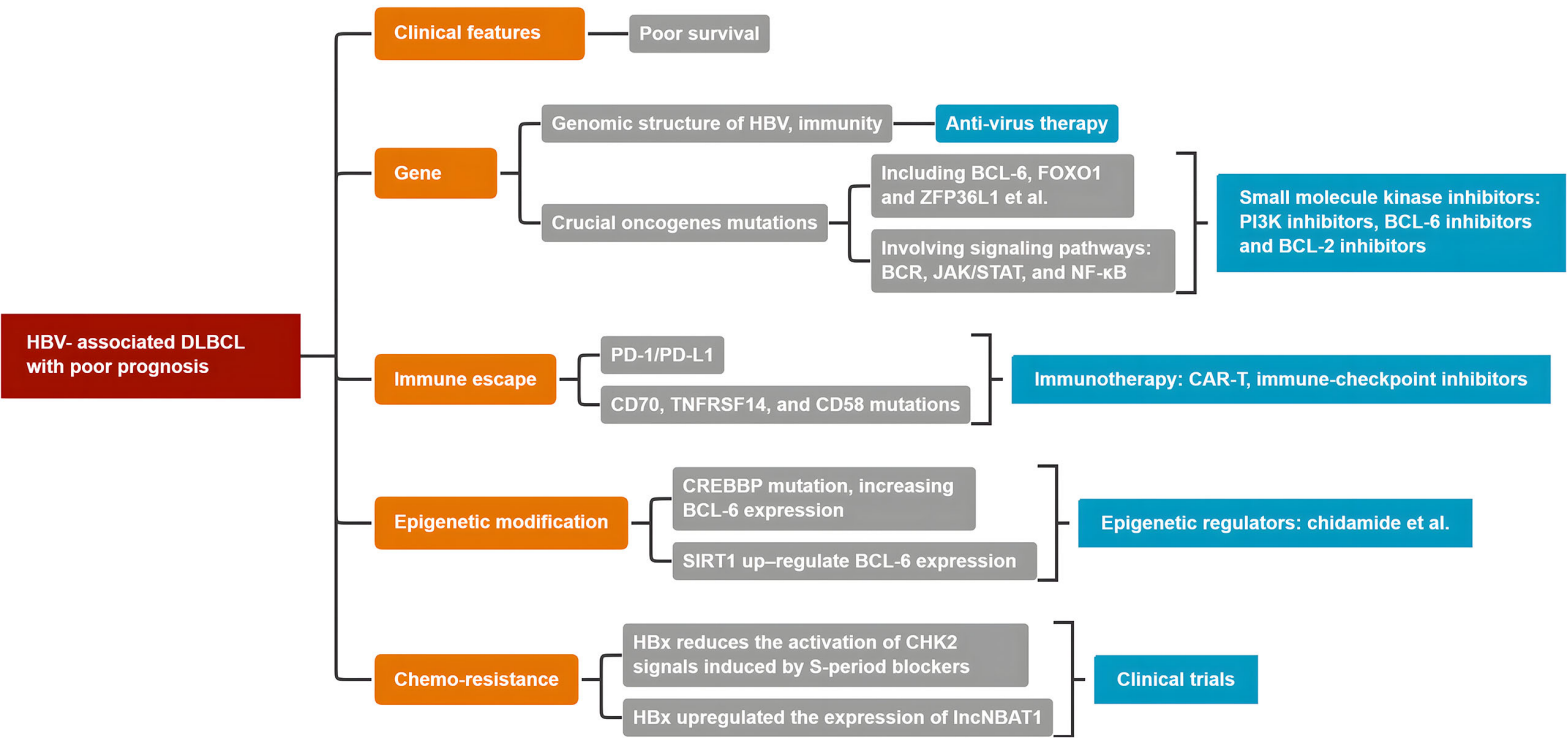
The conclusive algorithm of HBV-associated DLBCL.

Patients with HBV-associated DLBCL experience worse clinical prognosis and chemo-resistance. HBV infection and integration triggers inflammation favoring the accumulation of genetic and epigenetic lesions, involving many oncogenes, including crucial BCL-6, Myc, and CREBBP. Moreover, many signal pathways, including crucial BCR, JAK/STAT3, and the NF-κB pathway, are involved. Therefore, HBV, as a major risk factor, contributes to the development of HBV-associated DLBCL. There is no standard first-line immunochemotherapy in clinical settings. **Figure 5** presents a conclusive algorithm of crucial factors for HBV-associated DLBCL. Therefore, more clinical trials to implement personalized treatment approaches for HBV-associated DLBCL are warranted.

## Author contributions

XW, KY, and OB designed the study, provided vital data and wrote the manuscript. All authors contributed to the article and approved the submitted version.
